# *Lachnoclostridium*-mediated fermentation of *Konjac glucomannan*: short chain fatty acids production and inhibitory xanthine oxidase activity

**DOI:** 10.3389/fnut.2025.1690530

**Published:** 2025-12-17

**Authors:** Jie Deng, Yuxuan Liang, Zhiming Zhang, Zhiyi Yang, Yilu Bao, Yanyan Nong, Hongmei Wang, Chenhui Zhao, Shenghong Mai, Cong Tan, Yingxue Pan, Caimin Feng, Meiying Li, Wenfeng Luo

**Affiliations:** 1 Shunde Polytechnic University, Foshan, China; 2 Shenzhen Key Laboratory of Food Nutrition and Health, GuangDong Engineering Technology Research Center of Aquatic Food Processing and Safety Control, College of Chemistry and Environmental Engineering and Institute for Innovative Development of Food Industry, Shenzhen University, Shenzhen, China; 3 Central Laboratory of Panyu Central Hospital, The Affiliated Panyu Central Hospital of Guangzhou Medical University, Guangzhou, Guangdong, China; 4 Guangdong Provincial Key Lab of Food Safety and Quality, South China Agricultural University, Guangzhou, Guangdong, China

**Keywords:** *Konjac glucomannan*, *Lachnoclostridium*, short chain fatty acids, xanthine oxidase activity, uric acid

## Abstract

Hyperuricemia (HUA) is notably prevalent in various regions. In China, it has become the second most prevalent metabolic disorder. Since the adverse effects of conventional uric acid-lowering drugs, there is an urgent need to develop natural and safe therapeutic alternatives. Previous studies have shown that *Konjac glucomannan* (KGM) can effectively reduce serum uric acid levels in HUA rats and modulate gut microbiota composition, particularly by increasing the abundance of *Lachnoclostridium*. To further elucidate the underlying mechanisms, this study investigated the fermentation characteristics of KGM by *Lachnoclostridium* and its inhibitory effects on xanthine oxidase (XOD) activity. The results revealed that KGM fermentation with *Lachnoclostridium* reduced the pH and significantly lowered the apparent viscosity. Reducing sugar content decreased while short chain fatty acids (SCFAs) increased significantly. Furthermore, prolonged fermentation enhanced the XOD inhibitory activity of KGM. These findings suggested that KGM may exert its uric acid-lowering effects by promoting *Lachnoclostridium*-mediated fermentation of SCFAs, which may serve as foundation for the development of dietary strategies.

## 1 Introduction

Hyperuricemia (HUA) is a prevalent metabolic disorder characterized by impaired purine metabolism, which elevates serum uric acid levels and predisposes to complications such as gout and kidney dysfunction (1). Recent trends suggest a rising prevalence of HUA. Globally, the prevalence rate has been documented to range from 2.6 to 36% across various populations (2). According to the U.S. National Health and Nutrition Examination Survey, approximately 21% of adults, equating to 43 million individuals, have been diagnosed with HUA (3). Pharmacological treatments such as allopurinol and febuxostat effectively decrease uric acid level but pose risks of hepatic and renal toxicity with long-term use (4). Thus, there is an urgent need to explore safer, natural alternatives for HUA prevention and management. *Konjac glucomannan* (KGM) is a natural, water-soluble dietary fiber with diverse bioactivities (5). KGM has been shown to modulate intestinal microbiota and reduce serum uric acid levels in HUA rats (6). Microbiota-host co-metabolism plays a crucial role in maintaining uric acid homeostasis through complex regulatory mechanisms. The primary functions involved are the degradation of uric acid, regulation of its excretion, and suppression of systemic inflammation (7–9). Probiotics, such as *Lactobacillus*, have the capability to secrete uricase, an enzyme that facilitates the direct degradation of intestinal uric acid. Additionally, probiotics can enhance the expression of renal transporters, which contributes to the increased excretion of uric acid (10–12). It has been found that short chain fatty acids (SCFAs) biosynthesis by gut microbiota is markedly downregulated in HUA, which may exacerbate uric acid production (13). *Lachnoclostridium* is a key genus for SCFAs synthesis and shows a significant negative correlation with both diabetes and obesity (14). Clinical trials revealed that gut microbiota richness and diversity were significantly reduced in uric acid stone patients. Following potassium sodium hydrogen citrate treatment, serum uric acid decreased, while SCFAs-producing bacteria such as *Lachnoclostridium* were upregulated and SCFAs content markedly increased (15). Besides, *Lachnoclostridium* modulated acetic acid levels critical for cardiometabolic health (16). Furthermore, its metabolic activity also improved intestinal barrier function and antioxidant capacity by increasing SCFAs levels and activating G protein coupled receptors (17). Our earlier study demonstrated that KGM improved intestinal microbiota composition in HUA patient samples, significantly increased *Lachnoclostridium* abundance (18), suggesting that KGM may ameliorate HUA by modulating intestinal metabolite production via *Lachnoclostridium*. Based on these findings, this study investigates how *Lachnoclostridium* ferments KGM and whether the fermentation-derived metabolites inhibit xanthine oxidase (XOD), a key enzyme in uric acid synthesis.

## 2 Materials and methods

### 2.1 Determination of KGM molecular weight

KGM with 90% purity were provided by Huaxianzi Konjac Products Co., Ltd., (Hubei, China). The KGM samples were dissolved in 0.1 M NaN aqueous solution (containing 0.02% Na, w/w) to final concentration of 1 mg/mL and filtered through a 0.45 μm membrane for chromatographic analysis. The experiment was performed by a gel permeation chromatography-multiangle laser light scattering coupled system. Elution conditions were as follow: column temperature, 45 °C; injection volume, 100 μL; mobile phase A, 0.02% NaN in 0.1 M NaNO; flow rate, 0.5 mL/min; isocratic elution for 100 min. The molecular size information was obtained using a multiangle laser light scattering instrument, and molecular weight parameters were calculated based on the Mark-Houwink equation.

### 2.2 Preparation and inoculation fermentation of KGM solution

The *Lachnoclostridium* strain on the plate colony was purchased from Henan Provincial Engineering Research Center for Industrial Microbial Strains with the number BNCC379423. The complete fermentation medium was RCM medium contained (per liter) peptone (10 g), meat extract (10 g), yeast extract (3 g), glucose (5 g), soluble starch (1 g), L-cysteine hydrochloride (0.5 g), NaCl (5 g), sodium acetate (3 g), agarose (0.5 g), pH 6.8. KGM was added at 0.5% (w/v). Inoculation density was adjusted to 1 × 10^8^ CFU/mL. A 10 mL aliquot of RCM medium was transferred into a 14 mL shaking tube, to which 0.5% (w/v) KGM powder was added. KGM mixture was homogenized and subsequently placed in an anaerobic cultivation bag and subjected to deoxygenation at 4 °C for 24 h. *Lachnoclostridium* (5%) was introduced into the KGM solution and immediately homogenized with a vortex mixer, then incubated at 37 °C under anaerobic conditions for fermentation. The fermentation process was conducted for 0 h, 24 h and 48 h. Following fermentation, the broth was heat-inactivated, cooled to ambient temperature, and stored at 4 °C for subsequent use.

### 2.3 KGM fermentation broth pH and reducing sugar determination

Each KGM fermentation sample was centrifuged at 6,000 rpm and 4 °C for 5 min. The supernatant was subsequently collected for pH measurement. After the readings stabilized, the pH was recorded; each sample was measured in triplicate. Using a reducing sugar assay kit, glucose standards were prepared in quintuplicate by serial dilution: aliquots of 0.1–0.5 mL from a 1 mg/mL stock were brought to distilled water (0.5 mL). Eeach standard was mixed with 1 mL of DNS reagent, using distilled water as the blank. The samples were subjected to a boiling water bath for 5 min, and further diluted to a total volume of 4 mL. Absorbance was measured at 540 nm, and a glucose standard curve was constructed. KGM fermentation samples were centrifuged at 6000 rpm and 4 °C for 5 min; the supernatant was analyzed for reducing sugars, the content of which was calculated using the following Equation 1:


$$\text{Reducing sugar content}\;(\mathrm{mg}/g)=\frac{C\times D\times V_{T}}{W}$$ (1)

Among them, C, D and $V_{T}$ represent the concentration of the KGM fermentation, the dilution multiple and the total volume respectively, and W is the initial mass of the KGM fermentation substrate.

### 2.4 Determination of rheological properties of KGM fermentation

The rheological of the fermentation is critical parameter that reflect its flow and deformation behavior. In this study, a rotational rheometer (Anton Paar, Germany) was employed to measure the apparent viscosity of the KGM fermentation. Temperature was controlled at 25 °C via a precision water bath. The samples were loaded into the rheometer and dispersed. They were then subjected to continuous shear from 0 to 100 s^−1^ for 5 min.

### 2.5 SCFAs analysis

SCFAs levels were measured according to our previous study with certain modifications (19). The fermentation of KGM was subjected to centrifugation at 10,000 rpm for 20 min at 4 °C. Subsequently, 1 mL of the supernatant was collected and combined with 0.1 mL of 50% (v/v) sulfuric acid (H_2_SO_4_) and 1 mL of ethyl ether, followed by vortex mixing. The upper layer was then collected and filtered for SCFAs analysis. Transfer 50 μL of each standard, including acetic acid, propionic acid, butyric acid, valeric acid, and isovaleric acid (purity ≥ 99%, Sigma-Aldrich, USA) into a 10 mL volumetric flask and dilute to volume with diethyl ether. The calibration curves for the SCFAs cover the following concentration ranges: Acetic acid: 0.3–40 mmol/L; Propionic acid: 0.3–40 mmol/L; Butyric acid: 0.01–40 mmol/L; Valeric acid: 0.01–40 mmol/L; Isovaleric acid: 0.01–40 mmol/L. Gas chromatography (GC) analysis was conducted using the Agilent 6,890 N GC system equipped with a flame ionization detector. The column utilized was an FFAP elastic quartz capillary column with dimensions of 30 m × 0.25 mm × 0.25 μm. The temperature program commenced at an initial column temperature of 70 °C, which was maintained for 1 min, followed by an increase at 5 °C/min to 150 °C. High-purity nitrogen served as the carrier gas at 2 mL/min. The detector temperature was set to 280 °C, and the injection volume was 2 μL. All samples were analyzed in triplicate.

### 2.6 XOD inhibition rate determination

The XOD-inhibitory activity of the fermentation samples was assessed refer to the instruction of XOD enzymatic activity assay kit (Beijing Boxbio Science & Technology Co., Ltd.). Initially, reconstitute the XOD standard (50 U/mg) with phosphate-buffered saline (PBS) to 0.2 U/mL. Then, 20 μL of XOD solution and 20 μL of fermentation sample solution were mixed uniformly and incubated at 37 °C for 5 min. Add 1 mL of the working solution from the XOD enzymatic activity assay kit and incubated at 37 °C for 5 min. For the sample control, the XOD enzyme solution was replaced with PBS. For the positive control, the KGM fermentation was replaced with PBS. For the blank control, both the KGM fermentation solution and XOD solution were replaced with PBS. Finally, measured the absorbance at 290 nm and calculated the XOD inhibition rate according to the Equation 2:


$$\text{Inhibition rate}(\%)=(1-\frac{A_{\text{sample}}-A_{\text{sample control}}}{A_{\text{positive control}}-A_{\text{blank control}}})\times 100\%$$ (2)

### 2.7 Statistical analysis

GraphPad Prism was applied for data analysis, all experiments were performed with biological replicates, defined as independent fermentation runs, and results were presented as means ± SEM (*n* = 3). Significant differences were calculated by SPSS 20.0 software and analyzed using One-way ANOVA analysis followed by Tukey’s multiple comparison test (*p* < 0.05). Correlation matrix between the inhibition rate of XOD and SCFAs was performed by OriginPro through Pearson’s test.

## 3 Results and discussion

### 3.1 Molecular weight structure of KGM

**Figure 1:**
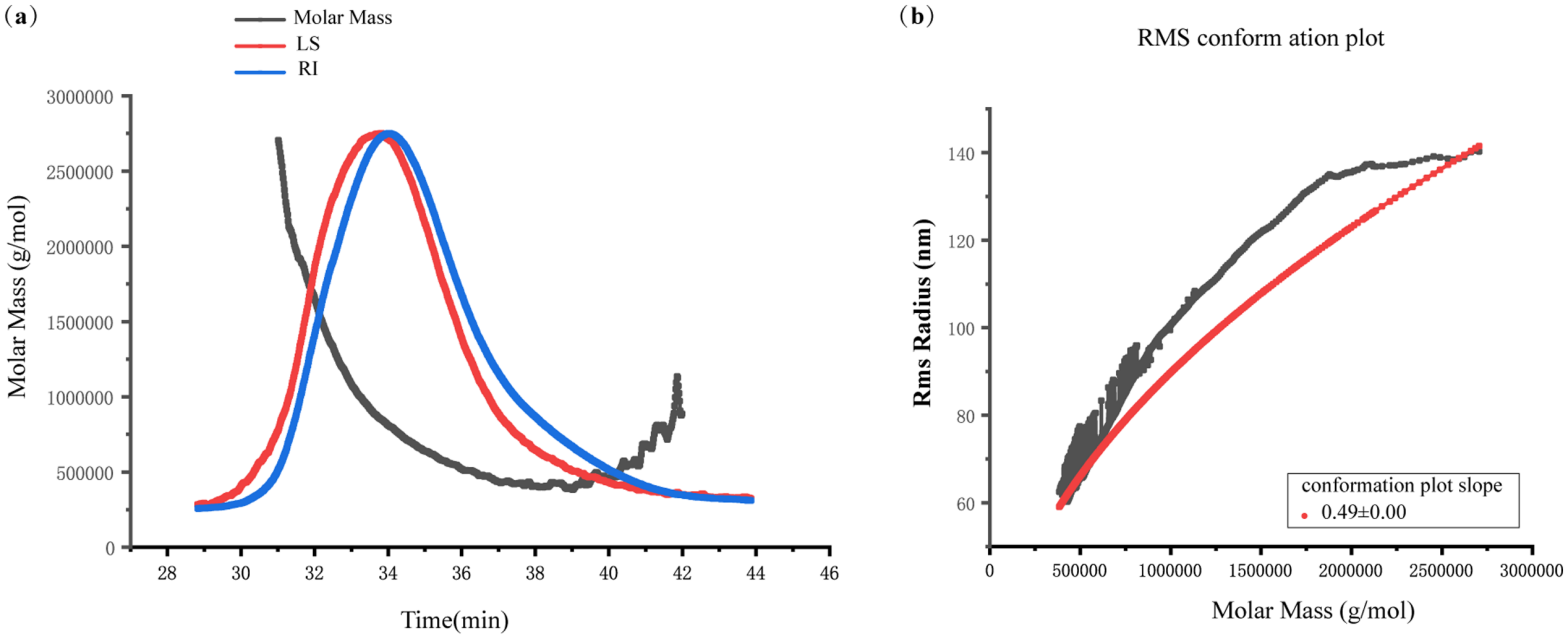
KGM molecular weight distribution and molecular conformation. **(a)** Absolute molecular weight analysis. **(b)** Molecular configuration analysis.

Figure 1a shows the molecular weight distribution curve of KGM, revealing an average molecular weight of 825.87 kDa and a root-mean-square radius of 81.4 nm. Figure 1b presents the molecular configuration diagram of KGM. Polysaccharide conformation, a critical structural feature, which can be assessed by the slope of the root-mean-square radius relative to the molar mass (20). Similarly to our previous study (21), this finding demonstrated that KGM adopts a random coil conformation characterized by irregular coiled shape.

### 3.2 Dynamics of pH and reducing sugar concentration in KGM fermented by *Lachnoclostridium*

**Figure 2:**
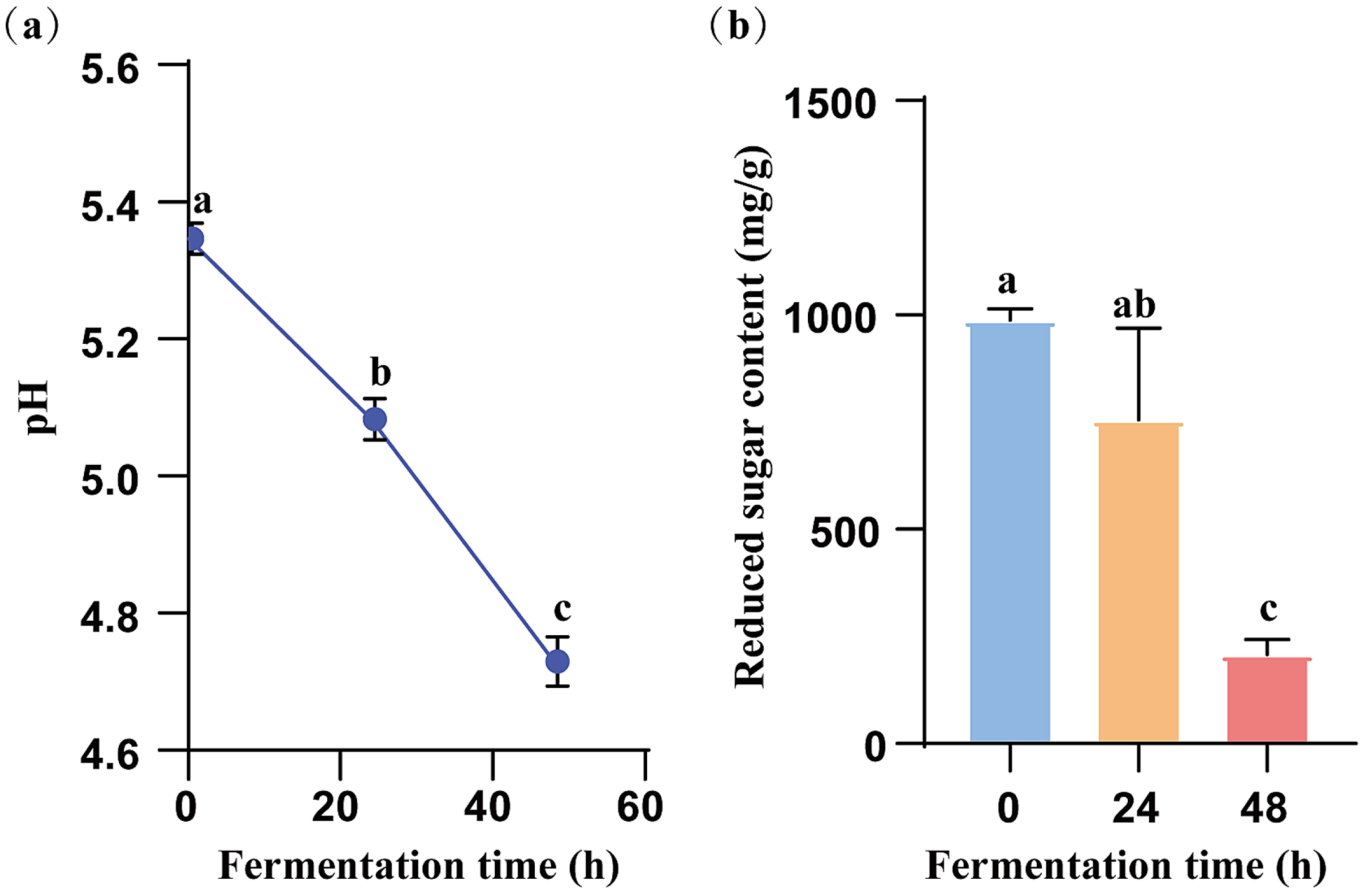
Value of pH and reducing sugar concentration of KGM fermentation at 0 h, 24 h and 48 h. **(a)** Value of pH. **(b)** Reducing sugar concentration. Data were expressed as means ± SEM (*n* = 3), there were significant differences between groups with different letters (*p* < 0.05).

The pH indicates fermentation progress, reflecting dissociated acid concentration (22). As illustrated in Figure 2a, the pH of the KGM fermentation medium decreased markedly over time, indicating continuous accumulation of acidic metabolites driven by microbial activity, which was consistent with former study (23). The initial pH of KGM-0 h was 5.35, whereas the pH values for *Lachnoclostridium*-fermented KGM-24 h and KGM-48 h were 5.08 and 4.73, respectively. These significant changes in pH indicated that KGM has been effectively fermented by *Lachnoclostridium*. Furthermore, Figure 2b showed reducing sugar content decreased over time, indicating that *Lachnoclostridium* may progressively consume KGM as a key carbon source.

### 3.3 Changes in the rheological properties of KGM fermentation

**Figure 3:**
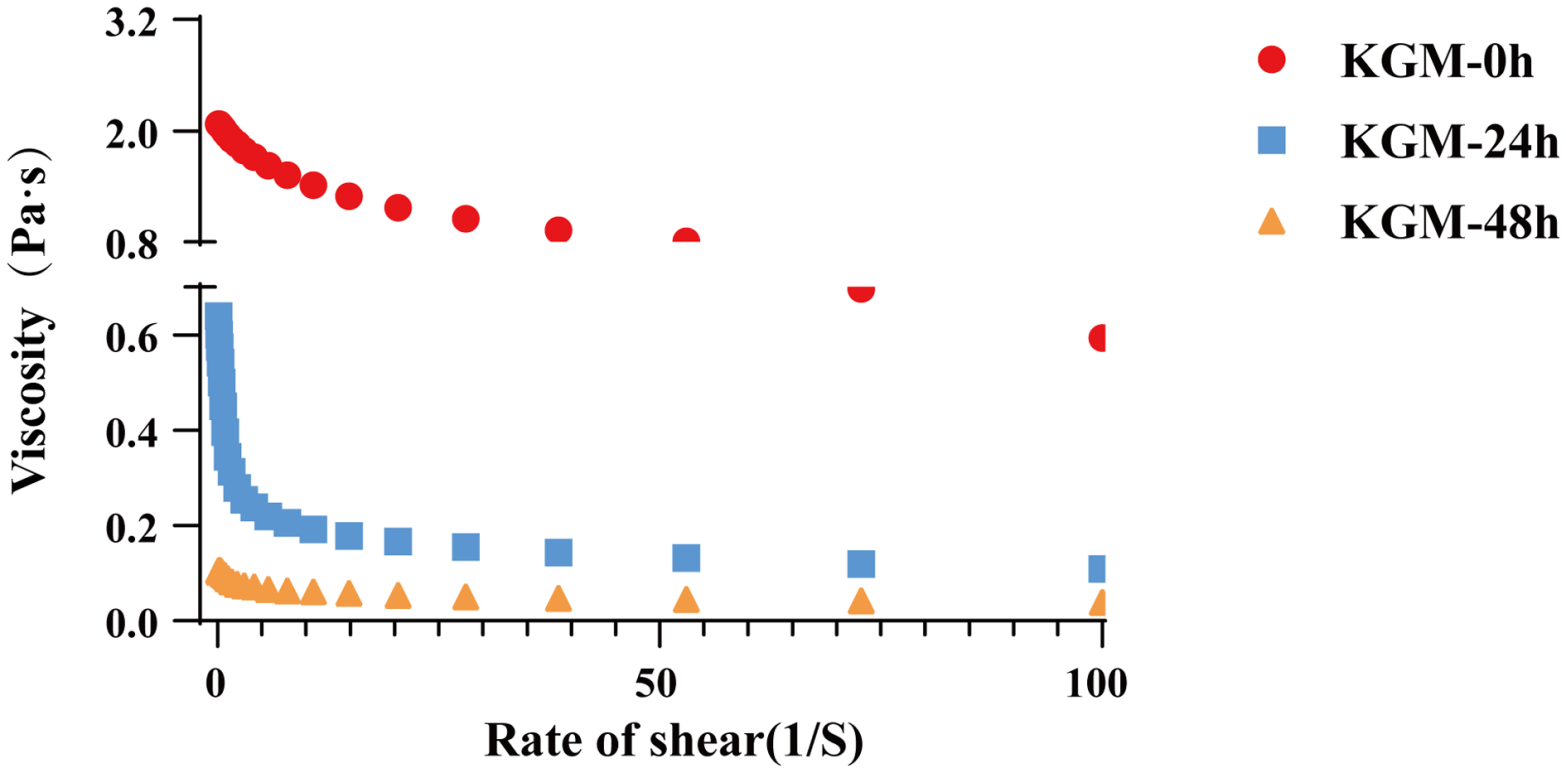
Viscosity characterization of KGM fermentation at 0 h, 24 h and 48 h.

Steady shear analysis of KGM fermentation broth at different stages revealed pronounced shear-thinning behavior: viscosity decreased markedly with increasing shear rate, particularly at low shear rates (24). As illustrated in Figure 3, the viscosity of the KGM-24 h and KGM-48 h fermentation is significantly reduced compared to the KGM-0 h. The KGM-48 h exhibited further significant decrease in viscosity relative to the KGM-24 h. This viscosity reduction suggests Lachnoclostridium may hydrolyze *β*-1,4 glycosidic bonds in KGM chains (25). Additionally, fermentation derived organic acids may disrupt the intermolecular hydrogen bond network (26).

### 3.4 Profile of SCFAs production during KGM fermentation by *Lachnoclostridium*

**Figure 4:**
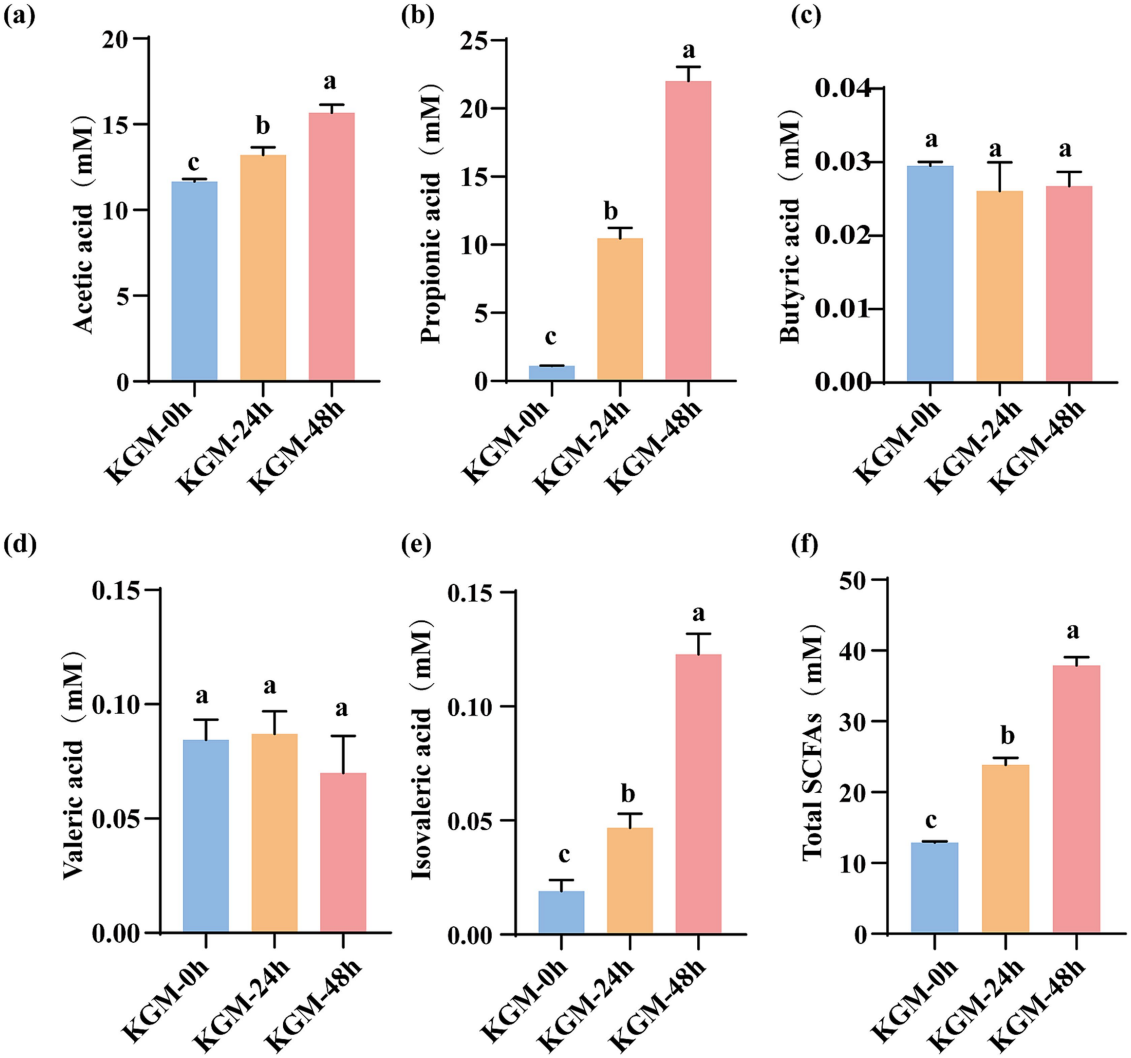
The levels of SCFAs in different groups after fermentation. **(a)** Acetic acid **(b)** propionic acid **(c)** butyric acid **(d)** valeric acid **(e)** isovaleric acid **(f)** total SCFAs. Data were expressed as means ± SEM (*n* = 3), there were significant differences between groups with different letters (*p* < 0.05).

SCFAs are crucial for intestinal health, immune regulation, and metabolic homeostasis (27). Research illustrated that dietary components and microbial metabolites modulate HUA through intestinal microbiota interactions, inhibiting XOD activity to suppress uric acid synthesis, and up-regulating urate transporters to accelerate uric acid excretion (28). This study demonstrated that *Lachnoclostridium*-mediated fermentation of KGM selectively produces SCFAs, dominated by acetic, propionic, butyric, valeric, and isovaleric acids. Figure 4 showed that acetic acid, propionic acid, and isovaleric acid concentrations increased markedly with prolonged fermentation time. When compared to the KGM-0 h, levels of acetic acid, propionic acid, isovaleric acid, and total SCFAs increased significantly in KGM-24 h and KGM-48 h (*p* < 0.05). However, the concentrations of butyric acid and valeric acid remained no significant change throughout fermentation, likely reflecting their specific metabolic synthesis pathways.

### 3.5 Changes in XOD inhibitory activity of *Lachnoclostridium*-fermented KGM

**Figure 5:**
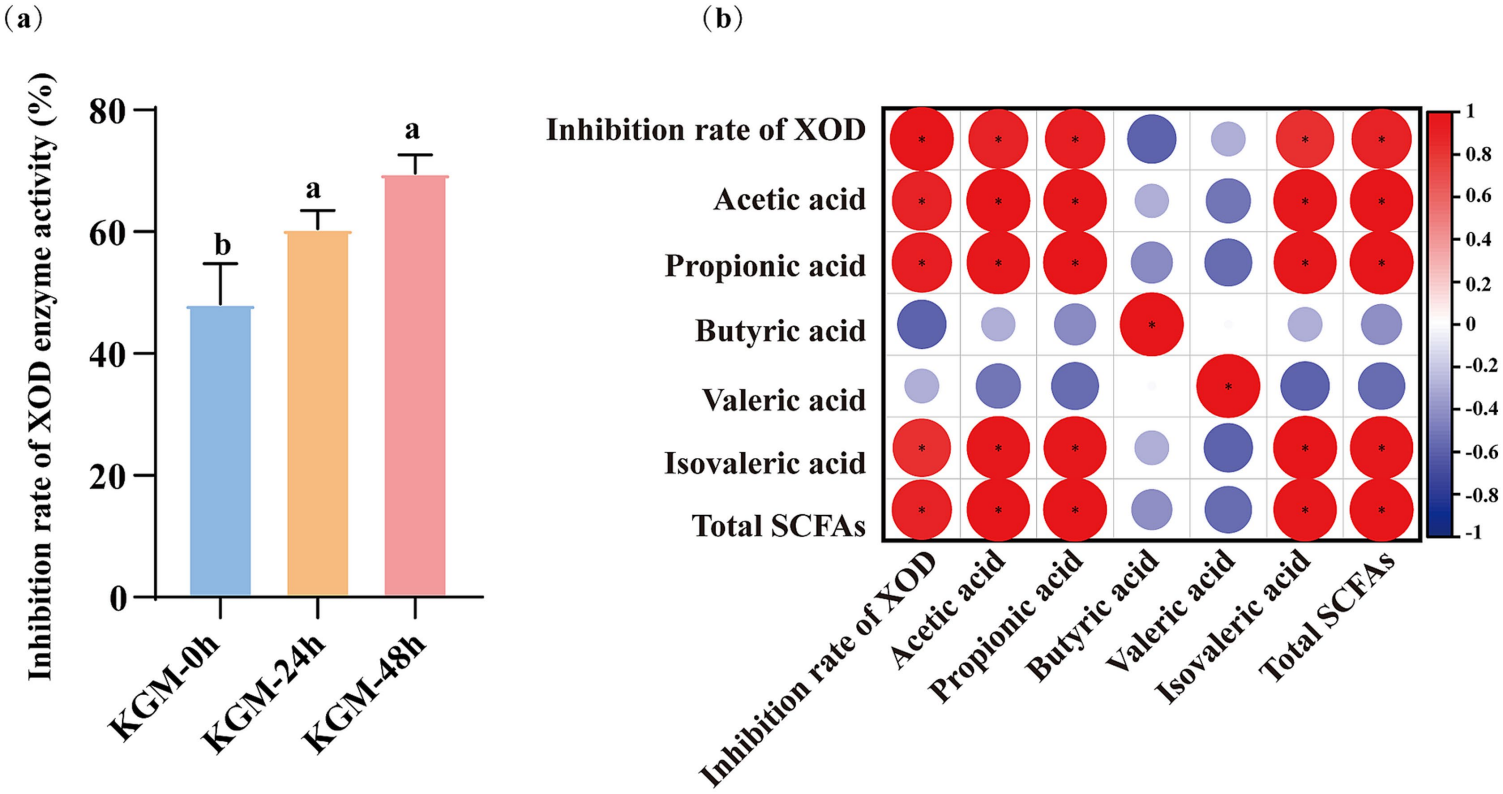
Effect of KGM fermentation products on inhibition rate of XOD enzyme activity and correlation analysis. **(a)** Inhibition rate of XOD enzyme activity. Data were expressed as means ± SEM (*n* = 3), there were significant differences between groups with different letters (*p* < 0.05). **(b)** Correlation matrix among SCFAs level and inhibition rate of XOD activity. Larger circle represent stronger correlations, with red and blue indicating positive and negative correlation, respectively. Asterisks (*) denoted statistical significance (*p < 0.05).

XOD, a key enzyme in uric acid synthesis, is predominantly distributed in mammalian liver and intestine (29). The enzyme catalyzes the oxidation of hypoxanthine to xanthine and directly catalyzes xanthine to uric acid (30). Studies have shown that natural substances can inhibit XOD activity and reduce serum uric acid level (31). As shown in Figure 5a, with KGM fermentation time increased, the inhibition rate of XOD shows an upward trend. The inhibition rate of XOD by KGM-0 h was 48.15%, suggesting that native KGM may have certain inhibitory effects on XOD. After 24 h of fermentation, the inhibition rate of KGM-24 h increased to 60.49%, and further elevated to 69.62% in KGM-48 h. This indicates that the inhibitory effect of KGM fermentation on XOD gradually intensified over time, suggesting the production of more potent inhibitory metabolites. The correlation analysis (Figure 5b) demonstrated significant relationships between XOD inhibition activity and acetic acid, propionic acid, isovaleric acid and total SCFAs (*p* < 0.05), but no significant difference was found in butyric acid and valeric acid. These findings suggested that SCFAs were associated with the enhanced XOD inhibition, but its specific causal role awaits further validation.

## 4 Discussion

This study determined that the molecular weight of KGM is 825.87 kDa, exhibiting a typical random coil conformation, which was consistent with previous study (21). The high molecular weight and branch structural features provided the physicochemical basis for its effective utilization by microorganisms (32). The random-coil conformation of KGM implied a high segmental mobility and extensive solvent-exposed surface area, which facilitates the hydrolytic enzymes secreted by *Lachnoclostridium* to access internal glycosidic bonds (19, 33). Conformably, the study observed a rapid decline in reducing sugars in the early stages of fermentation.

Further analysis showed that the main fermentation products were acetic acid, propionic acid, and isovaleric acid, while the changes in butyric acid and valeric acid were not significant. The findings align with the metabolic attributes of *Lachnoclostridium*, predominantly characterized by the synthesis of propionic acid, which probably via the acetyl-CoA → propionyl-CoA pathway (34, 35). The study suggested that the degradation products of KGM may preferentially promote the propionic acid synthesis pathway, whereas the butyric acid content did not increase significantly, which was similarly to previous study (36). From the perspective of health effects, propionic acid not only activated the G-protein-coupled receptors to regulate immune metabolism but also inhibited liver XOD expression, thereby exerting a uric acid-lowering effect (37). The inhibition rate of XOD by fermentation supernatant exhibited a notable increase from 48.15% at 0 h to 69.62% at 48 h, demonstrating a significant temporal correlation. Consistently, *Lactobacillus rhamnosus* R31, *L. rhamnosus* R28-1 and *L. reuteri* L20M3 markedly lowered serum and urinary UA levels and attenuated XOD activity of blood and liver in HUA mice. Correlation analysis indicated that these UA-reducing lactic acid bacteria boost SCFAs production by increasing SCFAs-producing microbiota, thereby suppressing XOD activity in serum and liver (38). In our study, while Pearson’s analysis indicated significant correlation between XOD inhibition activity and acetic acid, propionic acid, isovaleric acid and total SCFAs, this relationship is associative rather than causative. The fermentation broth is a complex mixture; therefore, the specific contribution of SCFAs cannot be clearly clarified by correlation alone. On the other hand, KGM fragments generated by *Lachnoclostridium* may act as competitive inhibitors at the XOD active. Previous study found that porphyra polysaccharide could spontaneously bind to XOD at a single class of hydrophobic sites, perturbing its secondary structure and plugging the catalytic cavity, thereby blocking enzyme activity and offering a new paradigm for natural XOD inhibition (39). Therefore, subsequent studies will incorporate allopurinol as positive control and isolate SCFAs at physiologically relevant concentrations to evaluate its direct inhibitory effect on XOD activity, thereby establishing causal efficacy.

## 5 Conclusion

In this study, the *in vitro* uric acid-lowering activity and metabolic products of KGM fermented by *Lachnoclostridium* were investigated. The results showed that the molecular conformation of KGM was an irregular coil of flexible polymer chains, and KGM likely degraded by *Lachnoclostridium*, accompanied with significant decrease in pH. Meanwhile, the apparent viscosity of KGM fermentation was greatly reduced. In addition, reducing sugar content decreased significantly during fermentation while total SCFAs content increased markedly, indicating efficient conversion of KGM-derived reducing sugars into SCFAs by *Lachnoclostridium*. SCFAs not only contribute to the regulation of intestinal microbiota homeostasis, but also may affect uric acid metabolism. *Lachnoclostridium* fermentation of KGM significantly enhanced XOD inhibitory activity, which indicated that KGM fermentation yields increasingly potent XOD inhibitors, such as SCFAs. These findings provide evidence supporting the uric acid lowering potential of KGM fermentation products and establish a basis for the utilization of KGM in promoting intestinal health and intervening in metabolic diseases. It is important to note that this study was conducted *in vitro* and focused on a single bacterial genus. Extrapolating these findings to *in vivo* HUA outcomes will require validation through animal models and clinical trials, accounting for the absorption, distribution, and systemic metabolism of SCFAs.

## Data Availability

The raw data supporting the conclusions of this article will be made available by the authors, without undue reservation.
